# Data on environmentally relevant level of aflatoxin B_1_-induced human dendritic cells' functional alteration

**DOI:** 10.1016/j.dib.2018.04.104

**Published:** 2018-04-30

**Authors:** Jalil Mehrzad, Abbas Bahari, Mohammad Reza Bassami, Mahmoud Mahmoudi, Hesam Dehghani

**Affiliations:** a Department of Microbiology and Immunology, Faculty of Veterinary Medicine, University of Tehran, Tehran, Iran; b Department of Pathobiology, Faculty of Veterinary Medicine, and Institute of Biotechnology, Ferdowsi University of Mashhad, Mashhad, Iran; c Department of Clinical Science, Faculty of Veterinary Medicine, and Institute of Biotechnology, Ferdowsi University of Mashhad, Mashhad, Iran; d Department of Immunology and Allergy, School of Medicine, Mashhad University of Medical Sciences, Mashhad, Iran; e Department of Basic Sciences, Faculty of Veterinary Medicine, and Institute of Biotechnology, Ferdowsi University of Mashhad, Mashhad, Iran

**Keywords:** AFB_1_, Apoptosis, AFB_1_-detoxifying genes, Dendritic cells, Flow cytometry, Functional genes, Immunnoderegulation, Phagocytosis, RT-qPCR

## Abstract

We assessed the effects of naturally occurring levels of AFB_1_ on the expression of key immune molecules and function of human monocyte-derived dendritic cells (MDDCs) by cell culture, RT-qPCR, and flow cytometry. Data here revealed that an environmentally relevant level of AFB_1_ led to remarkably weakened key functional capacity of DCs, up-regulation of key transcripts and DCs apoptosis, down-regulation of key phagocytic element, CD64, and creation of pseudolicensing direction of DCs. Flow cytometry data confirmed a damage towards DCs, i.e., increased apoptosis. The detailed data and their mechanistic effects and the outcome are available in this research article (Mehrzad et al., 2018) [1]. The impaired phagocytosis capacity with triggered pseudolicensing direction of MDDCs caused by AFB_1_ and dysregulation of the key functional genes could provide a mechanistic explanation for the observed *in vivo* immunotoxicity associated with this mycotoxin.

**Specifications table**

**Table:** 

Subject area	*Immunobiology, molecular biology and immunotoxicology*
More specific subject area	*Environmental-related pro-inflammation and immunotoxicity that potentially lead to immunodysregulation through dendritic cells (DCs)*
Type of data	*Very concise reader friendly graphs, figures, tables, text and file with 25 various immune genes and their designed sequences*
How data was acquired	*Cell culture, flow cytometry and RT-qPCR*
Data format	*Analyzed data with interpretive idea*
Experimental factors	*Naturally occurring levels of AFB*_*1*_*on mass generated DCs from pure monocytes of healthy individuals, the expression of key immune molecules and function of human MDDCs by cell culture, RT-qPCR, and flow cytometry.*
Experimental features	*in vitro experimental cell culture data and assays as detailed in the original article*
Data source location	*Laboratories of Immunology and Biotechnology, Department of Microbiology and Immunology and Department of Pathobiology, Faculty of Veterinary Medicine, and Institute of Biotechnology, University of Tehran and Ferdowsi University of Mashhad, Iran*
Data accessibility	*Data provided in this article is usefully accessible to the public*

**Value of the data**

● The novel experimental model is key for further *in vitro* tests in the area of Immunology, immunobiology, molecular diagnosis and immunotoxicology.
● Provision of primary pure MDDCs and their 25 more key genes' sequences can be a road for finding the etiology of various infectious/non-infectious diseases in human.
● The immunotoxic aspects of environmental mycotoxins especially AFB_1_ and the key functional genes of MDDCs were designed as a model system for others to do further novel experiments in the area of mycotoxins-related infectious/non-infectious diseases, especially cancer.

## 1 Data

The analyses of the data on the expression of gene families in MDDCs indicated that the transcript levels of: 1) some key functional gene families, 2) some key TLR-related genes, 3) genes involved in the function of MDDCs and 4) some key cytokine transcripts were altered in post AFB_1_-exposed MDDCs. Further, the flow cytometry-based phagocytosis and apoptosis assay revealed diminished phagocytic and survival capacity of MDDCs. The main finding of data on immune molecules at mRNA levels is briefed in Table 1.

**Table 1:** An overview of the effects of AFB_1_ on the expression of various gene families and functional alterations in dendritic cells (DCs). The values (changes) are arbitrarily addressed according to the data in this article [1]. –=unchanged, ↑=increased, ↑↑=strongly increased, ↓=decreased, ↓↓=strongly decreased.

**Genes**	Arbitrary changes
**Aflatoxin B**_**1**_**metabolism genes**	
AhR	↑
AKR7A2	–
CYP1A2	↓
CYP1B1	↑
CYP3A4	↓
GSTM1	–
**TLRs-related genes**	
TLR2	↑↑
TLR4	↑↑
MyD88	↑↑
COX2	↑
NF-κb	–
**DC functional genes**	
CD64	↓↓
LFA3	↑
HLA-DR	↑↑
CD209	↑
CD11c	↓↓
CD16	↑
C5aR	–
CCR7	↑
**DC cytokines genes**	
TNF-α	↑↑
IL-6	–
IL-8	–
IL-1β	–
TGF-β	↓
IL-10	–
**DC phagocytic capacity**	↓↓
**DC apoptosis**	↑

**Fig. 1:**
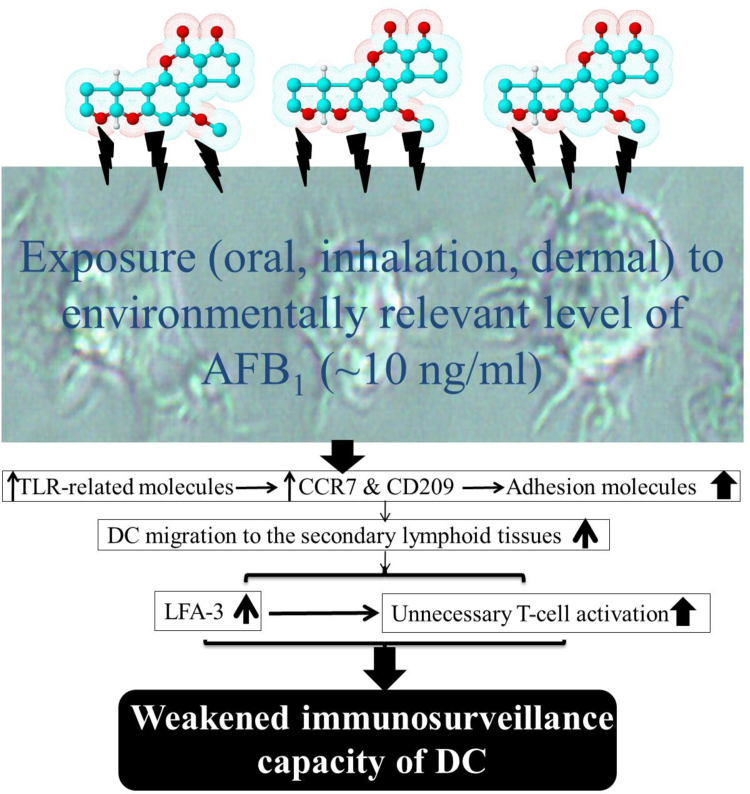
Schematic representation from the conceptual interpretation of data of this paper. The scheme depicts how the proposed issue of pseudolicensing of AFB_1_-exposed monocyte-derived dendritic cells (MDDCs) creates/leads to immunodisregulation *in vivo*. Though mRNA expression of CCR7, CD209 and LFA increase/change, nonetheless the protein levels of those molecules should be evaluated for future work.

Our data briefly illustrate (Fig. 1) the impaired phagocytosis capacity with triggered pseudolicensing [2] of MDDCs caused by AFB_1_ and dysregulation of the key functional genes could provide a mechanistic explanation for the observed *in vivo* immunotoxicity associated with this mycotoxin [3], [4], [5], [6], [7]. Here, we assessed >25 key molecules of AFB_1_-exposed MDDCs at mRNA level with some DCs’ key functional assays. Therefore, more in-depth molecular study at protein levels is needed.

## 2 Experimental design, materials and methods

Very briefly, experimental work plan were done on pure immature MDDCs with long membrane protrusions; accordingly, the MDDCs were treated with 0 or 10 ng of AFB_1_/ml for 2 and 12 h (37 °C, 5% CO_2_, 95% humidity) [3], [6] and then used for cellular and molecular analyses [3], [4], [5], [6], [7], [8]. Key functional gene families, TLR-related genes, genes involved in the function of MDDCs and some key cytokine transcripts were analyzed with qPCR assays [4], [5], [6], [8]. Further the flow cytometry assays were used to quantify phagocytosis and survival capacity of MDDCs [3], [6]. All data were as the mean±SEM of 8 experiments.
