# Supplementary material for: Data on environmentally relevant level of aflatoxin B1-induced human dendritic cells' functional alteration
Source: Data Brief. 2018 Apr 30;18:1576–80. doi: 10.1016/j.dib.2018.04.104 (PMC5999520; doi:10.1016/j.dib.2018.04.104)
Supplement: Supplementary file 1 — Supplementary material [file mmc1.docx]

**Conflict of interest**

None declared.
